# Measurement Uncertainty Estimation of a Robust Photometer Circuit

**DOI:** 10.3390/s90403149

**Published:** 2009-04-24

**Authors:** Wilmar Hernandez, Jesús de Vicente

**Affiliations:** 1 Department of Circuits and Systems in the EUIT de Telecomunicación at the Universidad Politécnica de Madrid (UPM), Campus Sur UPM, Ctra. Valencia km 7, Madrid 28031, Spain; 2 Department of Applied Physics in the ETSI Industriales at the Universidad Politécnica de Madrid, Calle José Gutierrez Abascal 2, Madrid 28006, Spain ; E-Mail: jvicente@etsii.upm.es; Tel.: +3-491-336-3125; Fax: +3-491-336-3000

**Keywords:** Photometer circuit, closed-loop transfer function, input quantity, uncertainty

## Abstract

In this paper the uncertainty of a robust photometer circuit (RPC) was estimated. Here, the RPC was considered as a measurement system, having input quantities that were inexactly known, and output quantities that consequently were also inexactly known. Input quantities represent information obtained from calibration certificates, specifications of manufacturers, and tabulated data. Output quantities describe the transfer function of the electrical part of the photodiode. Input quantities were the electronic components of the RPC, the parameters of the model of the photodiode and its sensitivity at 670 nm. The output quantities were the coefficients of both numerator and denominator of the closed-loop transfer function of the RPC. As an example, the gain and phase shift of the RPC versus frequency was evaluated from the transfer function, with their uncertainties and correlation coefficient. Results confirm the robustness of photodiode design.

## 1. Introduction

In general, there are many parameters that may affect a measurement result. Although it is impossible to identify all of them, the most significant can usually be identified and the magnitude of their respective effects on the measurement result can be estimated. Further, the way they impact the measurement result can, in many cases, be mathematically modeled [1].

In this paper, the uncertainty of measurement of a robust photometer circuit (RPC) based on both positive and negative feedback compensations was estimated. A rapid communication about the performance of the RPC was presented in [2]. Also, a detailed explanation of the electronic design of the RPC was given in [3]. The exact closed-loop transfer function (CLTF) of this complex feedback-controlled system was given in [4], a noise voltage analysis of it was carried out in [5] and an input-out transfer function analysis was carried out in [6].

In the above-mentioned references the importance of applying robust control techniques [7,8] to improve the disturbance rejection performance of photometer circuits was demonstrated. In addition, general information about signal conditioning and photodiode monitoring with operational amplifiers (opamps) by using non-robust feedback control techniques can be found in [9–11]. Other applications of robust and optimal filtering and control techniques to improve the performance of sensors can be found in [12–23].

The knowledge of the photodiode transfer function allows estimation of the RPC input from a measurement of its output. However, without an accompanying statement of the estimated uncertainty of RPC input, results are incomplete and in order to estimate the RPC input uncertainty, some estimation of the transfer function uncertainty is needed. The uncertainty of the measurement is a non-negative parameter characterizing the dispersion of the quantity values being attributed to the measurands based on the information used [24].

The aim of this paper is to estimate the uncertainty of the RPC transfer function (at a level of confidence of approximately 95% [25]) and show how from this information it is possible to estimate other RPC parameters, such as its gain and phase response, with their respective uncertainties. The description of the RPC transfer function is made through the coefficients of both numerator and denominator of this function.

## 2. CLTF of the RPC

**Figure 1.**
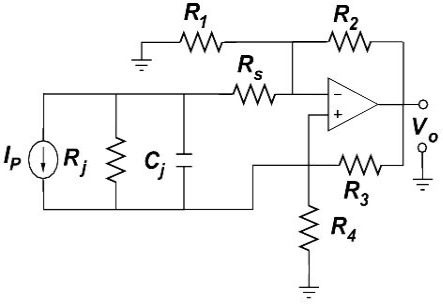
Robust photometer circuit.

In accordance with [2–5], the RPC is shown in Figure 1. Note that in this figure the photodiode diode has been substituted by its circuit model, which according to [9–11], among other references, consists of a current generator (*I_P_*) proportional to the incident light intensity, a junction capacitance (*Cj*), a shunt resistance (*Rj*), and a series resistance (*Rs*). Also, in this figure, *R*1, *R*2, *R*3 and *R*4 are the feedback resistors previously calculated in [3] that guarantee the robust disturbance rejection performance characteristic of the photometer circuit.

Therefore, taking into consideration opamp parameters such as the input resistance (*R_i_*), the input capacitance (*C_i_*), the open-loop gain (*A_o_*) and the gain bandwidth product (*w_T_* = 2π*f_T_*)), the CLTF from the current generator *i_P_*(*t*) to the output voltage *v_o_*(*t*) is given by:

(1) $$T_{1}\left(s\right)=\frac{L[v_{o}(t)](s)}{L[i_{P}\;(t)](s)}=\frac{R_{j}}{R_{j}+R_{s}}\cdot \frac{n_{1}(s)}{\left(s\cdot C_{j}\cdot R_{j}||R_{s}+1)\cdot d_{1}(s\right)}$$

where *R_j_* || *R_s_* is the parallel equivalent of *R_j_* and *R_s_*, and:

$$\begin{array}{c} n_{1}\left(s\right)=a_{3}s^{3}+a_{2}s^{2}+a_{1}s+a_{0}\\ d_{1}\left(s\right)=d_{5}s^{5}+d_{4}s^{4}+d_{3}s^{3}+d_{2}s^{2}+d_{1}s+d_{0} \end{array}$$

where all the coefficients of *n*_1_(*s*) and *d*_1_(*s*) have been deduced in [4]. In [4] the equation that describes the CLTF of the RPC as a function of the above opamp parameters was shown along with the stability analysis of the feedback system and some simulations and experimental results.

Here *s* = *j*ω (where $j=\sqrt{-1}$ and ω represents angular frequency), *L*[*v_o_*(*t*)](*s*) is the Laplace transform of the output voltage *v_o_*(*t*) and *L*[*i_P_*(*t*)](*s*) is the Laplace transform of the current *i_P_*(*t*).

Thus, taking into consideration (1), the CLTF from the power of the incident light *W*(*t*) to the output voltage *v_o_*(*t*) is given by:

(2) $$T_{2}\left(s\right)=\frac{L[v_{o}(t)](s)}{L[W(t)](s)}=T_{1}\left(s\right)\cdot \sigma (\lambda )$$

where σ(λ) is the sensitivity of the photodiode at a specific wavelength λ and *L*[*W*(*t*)](*s*) the Laplace transform of *W*(*t*).

From the above equations, it can be seen the influence of several aspects that are usually of concern for circuit designers such as operational amplifier parameters. For the problem at hand, the opamp parameters that have been taken into consideration to obtain the above equations are the ones that often limit the performance of photometer circuits based on opamps [4].

## 3. Applications of the Law of Propagation of Uncertainty

The law of propagation of uncertainty given in [24–25] assumes that the output quantity can be represented by a real number *y*, so that it can be written as a function that depends on one or more input quantities (i.e. x_1_, x_2_,⋯, x_m_)). The measurement function is given by:

$$y=f\left(x_{1},x_{2},\cdots ,x_{m}\right)$$

However, if there are *n* output quantities, the relation between the input and output quantities is given by:

y=f(x)

where $x={\left[\begin{array}{ccc} x_{1} & ... & x_{m} \end{array}\right]}^{T}$ (where the superscript ⊤ denotes transposition) and:

$$y=\left[\begin{array}{c} y_{1}\\ \vdots \\ y_{n} \end{array}\right]=\left[\begin{array}{c} f_{1}\left(x_{1},\cdots ,x_{m}\right)\\ \vdots \\ f_{n}\left(x_{1},\cdots ,x_{m}\right) \end{array}\right].$$

Furthermore, the uncertainty matrix of the vector x is given by:

$$U_{x}=\left[\begin{array}{ccc} u^{2}\left(x_{1}\right) & \cdots & u\left(x_{1},x_{m}\right)\\ \vdots & \ddots & \vdots \\ u\left(x_{m},x_{1}\right) & \cdots & u^{2}\left(x_{m}\right) \end{array}\right]$$

where *u*(*x_i_*) is the standard uncertainty of the input quantity *x_i_* and *u*(*x_i_*, *x_j_*) = *u*(*x_j_*, *x_i_*) is the estimated covariance of the input quantities *x_i_* and *x_j_*. The degree of correlation between *x_i_* and *x_j_* is characterized by the estimated correlation coefficient:

$$r\left(x_{i},x_{j}\right)=\frac{u\left(x_{i},x_{j}\right)}{u\left(x_{i}\right)\cdot u\left(x_{j}\right)}$$

where *r*(*x_i_, x_j_*) = *r*(*x_j_, x_i_*) and −1 ≤ *r*(*x_i_, x_j_*) ≤1. If the estimates *x_i_* and *x_j_* are independent of each other, *r*(*x_i_, x_j_*) = 0, and a change in one does not imply an expected change in the other.

In addition, the function y = f(x) is linearized at x = x_0_ and:

$$y=y_{0}+\Delta y=f\left(x_{0}+\Delta x\right)≅y_{0}+J\cdot \Delta x$$

where $y_{0}=f\left(x_{0}\right)={\left[\begin{array}{ccc} y_{10} & ... & y_{n0} \end{array}\right]}^{T}$, Δy = y – y_0_, $x_{0}={\left[\begin{array}{ccc} x_{10} & ... & x_{m0} \end{array}\right]}^{T}$, Δx = x – x_0_ and J is the Jacobian matrix of f(x):

$$J=\left[\begin{array}{ccc} \frac{{\partial f}_{1}}{{\partial x}_{1}} & \cdots & \frac{{\partial f}_{1}}{{\partial x}_{m}}\\ \vdots & \ddots & \vdots \\ \frac{{\partial f}_{n}}{{\partial x}_{1}} & \cdots & \frac{{\partial f}_{n}}{{\partial x}_{m}} \end{array}\right]$$.

Thus, the uncertainty matrix of the vector y is given by U_y_ = J · U_x_ · J*^T^* [27].

The elements *∂f_i_*/*∂x_j_* of the Jacobian matrix J are the sensitivity coefficients *c_ij_* of the output quantities *y_i_* associated to the input quantities *x_j_*. In this paper, in order to build matrix J numerical differentiation was used [28].

## 4. Results of the Experiment

### 4.1. Uncertainty of the Input Quantities and Typical Value of the CLTF

According to [26], input quantities represent information obtained from sources such as direct measurements, calibration certificates, specifications of manufacturers, and tabulated data. Table 1 shows the minimum, typical and maximum value of the input quantities, and their standard uncertainties as well.

The information of the parameters of the OP07 and the junction capacitance of the BPW21 was taken from their datasheets. The value of the resistors *R*_1_ – *R*_4_ were the nominal ones, the series resistance and the shunt resistance of the BPW21 were measured experimentally by using the KEITHLEY Semiconductor Characterization System 4200-SGS, and the sensitivity of the BPW21 was measured experimentally by using the 3 mW RS Modulated Laser Diode Module 194-004 at 0 Hz and nominal wavelength 670 nm. A photograph of the prototype of the RPC with the 3 mW Modulated Laser Diode Module was shown in [4].

In accordance with [24,25], taking into consideration the available information concerning the input quantities, in this paper the input quantities were described by triangular a priori distributions. Finally, using the above typical values and taking into consideration that *C_i_* = 0 for the OP07, the CLTF of the RPC given by (2) was given by:

(3) $$T_{2}(s)=\sigma (\lambda )\cdot \frac{n_{2}(s)}{d_{2}(s)}$$

Where:

$$\begin{array}{c} n_{2}(s)=0\cdot s^{3}+p_{1}s^{2}+p_{2}s+p_{3}\\ d_{2}(s)=0\cdot s^{6}+0\cdot s^{5}+q_{1}s^{4}+q_{2}s^{3}+q_{3}s^{2}+q_{4}s+q_{5} \end{array}$$

When C_i_ = 0, coefficients a_3_, d_4_ and d_5_ of (1) are equal to zero (see [4]). Therefore, n_1_(s) is a second order polynomial and d_1_(s) is a third order polynomial. Thus, in (3) the first term of the numerator, n_2_(s), is equal to zero and the first two terms of the denominator, d_2_(s), are equal to zero as well.

In order to have dimensionless parameter when possible the following change in polynomial *n*_2_(*s*) and *d*_2_(*s*) was made:

$$T_{2}(s)=y_{1}\cdot \frac{y_{2}\cdot {\left(s/w_{0}\right)}^{2}+y_{3}\cdot (s/w_{0})+1}{y_{4}\cdot {\left(s/w_{0}\right)}^{4}+y_{5}\cdot {\left(s/w_{0}\right)}^{3}+y_{6}\cdot {\left(s/w_{0}\right)}^{2}+y_{7}\cdot (s/w_{0})+1}$$

where *w*_0_ is a conventional value *w*_0_ = 1.5 *Mrad*/*s*. In this paper, the conventional value *w*_0_ has been chosen to be equal to the nominal value of the gain bandwidth product *w_T_* of the operational amplifier.

Please note that a conventional value has no uncertainty. Working in this way, the parameters *y*_2_ to *y*_7_ are dimensionless and the parameter *y*_1_ (the RPC gain at DC) is expressed in V/W. The expressions that relate the parameters *y_i_* with coefficients *p_j_* and *q_k_* of polynomials *n*_2_(*s*) and *d*_2_(*s*) are the following:

$$\begin{array}{cc} \begin{array}{l} y_{1}=p_{3}/q_{5}\cdot \sigma (\lambda )\\ y_{2}=p_{1}/p_{3}\cdot w_{0}^{2}\\ y_{3}=p_{2}/p_{3}\cdot w_{0} \end{array} & \begin{array}{l} y_{4}=q_{1}/q_{5}\cdot w_{0}^{4}\\ y_{5}=q_{2}/q_{5}\cdot w_{0}^{3}\\ y_{6}=q_{3}/q_{5}\cdot w_{0}^{2}\\ y_{7}=q_{4}/q_{5}\cdot w_{0} \end{array} \end{array}$$

The first parameter *y*_1_ can be easily determined by direct calibration: a power stabilized laser, whose power *W_C_* has been previously measured by a traceable laser power meter, is focused onto the photodiode and the output voltage of the RPC is measured with a traceable voltmeter. The reading provided by the voltmeter is *V_C_*, and an estimation of the RPC gain *y*_1_ at DC would be *g_DC_* = *V_C_*/*W_C_* with standard uncertainty *u*(*g_DC_*):

$$u(g_{\mathrm{DC}})=g_{\mathrm{DC}}\cdot \sqrt{{\left[u(V_{C})/V_{C}\right]}^{2}+{\left[u(W_{C})/W_{C}\right]}^{2}}$$

At this point it should be pointed out that as we are carrying out a direct calibration procedure, due to the fact that *g_DC_* has no relation with the estimations *y*_2_, *y*_3_, ⋯, *y*_7_, the covariance *u*(*g_DC_*) is equal to zero for *y_i_*, *i* > 1.

Thus, the RPC transfer function is described by using the output quantities *g_DC_* ≡ *y*_1_, *y*_2_, *y*_3_, ⋯, *y*_7_. This transfer function allows us to carry out the estimation of the power of the optical signal *W*(*t*) arriving at the photodiode, through the measurement of the electrical output signal *v*_0_(*t*):

$$W(t)=L^{-1}\left\{\frac{L[v_{0}(t)](s)}{T_{2}(s)}\right\}\;(t)$$

As in this paper the photodiode is operated in the photoconductive mode, the photocurrent is linearly proportional to the incident light energy. Thus, assuming we have no nonlinear distortion in the opamp, the RPC shown in Figure 1 is a linear circuit; and for the case in which the optical signal is harmonic, *W*(*t*) = *W*_0_cos(*ωt* + *α*), the output voltage is harmonic as well, *v*_0_(*t*) = *V*_0_cos(*ωt* + φ + *α*), where *ω* and α are the angular frequency and phase shift of the optical signal, respectively.

The amplitude *V*_0_ of the output voltage is determined by:

$$\frac{V_{0}}{W_{0}}=G(\omega )=\left|T_{2}(j\omega )\right|.$$

The gain *G*(ω) can be expressed as *G*(ω) = *g_DC_* · *g*(ω), where *g*(ω) is:

$$g(\omega )=\left|\frac{y_{2}\cdot {\left(j\omega /w_{0}\right)}^{2}+y_{3}\cdot (j\omega /w_{0})+1}{y_{4}\cdot {\left(j\omega /w_{0}\right)}^{4}+y_{5}\cdot {\left(j\omega /w_{0}\right)}^{3}+y_{6}\cdot {\left(j\omega /w_{0}\right)}^{2}+y_{7}\cdot (j\omega /w_{0})+1}\right|$$

And the phase shift φ is determined by:

$$\varphi (\omega )=\text{arg}\left(T_{2}(j\omega )\right)=\text{arg}\left(\frac{y_{2}\cdot {\left(j\omega /w_{0}\right)}^{2}+y_{3}\cdot (j\omega /w_{0})+1}{y_{4}\cdot {\left(j\omega /w_{0}\right)}^{4}+y_{5}\cdot {\left(j\omega /w_{0}\right)}^{3}+y_{6}\cdot {\left(j\omega /w_{0}\right)}^{2}+y_{7}\cdot (j\omega /w_{0})+1}\right)$$

### 4.2. Uncertainty of the Parameters Describing the Transfer Function

The standard uncertainty and the relative standard uncertainty (which is defined as the ratio of the standard uncertainty of the parameter to its typical value) of the parameters *y*_2_, *y*_3_, ⋯, *y*_7_, are shown in Table 2.

The matrix of the estimated correlation coefficients among elements of parameters *y*_2_, *y*_3_, ⋯, *y*_7_ is:

$$R=\left[r\left(x_{i},x_{j}\right)\right]=\left[\begin{array}{cccccc} 1 & 0.98 & -0.05 & -0.16 & -0.33 & 0.38\\ 0.98 & 1 & -0.17 & -0.24 & -0.39 & 0.29\\ -0.05 & -0.17 & 1 & 0.96 & 0.88 & 0.64\\ -0.16 & -0.24 & 0.96 & 1 & 0.95 & 0.58\\ -0.33 & -0.39 & 0.88 & 0.95 & 1 & 0.47\\ 0.38 & 0.29 & 0.64 & 0.58 & 0.47 & 1 \end{array}\right].$$

where *r*(*x_i_*, *x_j_*) = *r*(*y_i_*, *y_j_*) and i = 2,3 and j = 4,5,6,7.

At first glance, *y*_2,3_ seem to be no correlated with *y*_4,5,6,7_ because they depend on different variables, *y*_2,3_ depend on *p*_1,2,3_ while *y*_4,5,6,7_ depend on *q*_1,2,3,4,5_. However, as *p*_1,2,3_ are correlated with *q*_1,2,3,4,5_, *y*_2,3_ and *y*_4,5,6,7_ are correlated as well.

The uncertainty matrix U*_y_* of the parameters *y*_2_, *y*_3_, ⋯, *y*_7_ is:

$$U_{y}=\left[\begin{array}{cccc} u^{2}(y_{2}) & r_{23}u(y_{2})u(y_{3}) & \cdots & r_{27}u(y_{2})u(y_{7})\\ r_{32}u(y_{3})u(y_{2}) & u^{2}(y_{3}) & & r_{37}u(y_{3})u(y_{7})\\ \vdots & & \ddots & \vdots \\ r_{72}u(y_{7})u(y_{2}) & r_{73}u(y_{7})u(y_{3}) & \cdots & u^{2}(y_{7}) \end{array}\right].$$

### 4.3. Using the Transfer Function to Determine Other Parameters: Gain, Phase Shift and Cut-Off Frequency of the RPC

As described previously, the transfer function can be used to determine the gain *G*(ω) = *g_DC_* · *g*(*ω*) and the phase shift φ(ω) of the RPC when the optical power arriving at the photodiode is harmonic. The angular frequency of the optical signal can be expressed as *ω* = 2π*f*, where *f* is its frequency.

#### 4.3.1. Gain and Phase Shift

Again, the uncertainty propagation from the transfer function parameters *g_DC_* ≡ *y*_1_, *y*_2_, *y*_3_, ⋯, *y*_7_ to *G*(ω) = *g_DC_* · *g*(*ω*) and φ(*ω*) are calculated by using the procedure described in Section 3. At a specified frequency *f*, the gain and phase shift are functions of the parameters *y*_2_, *y*_3_, ⋯, *y*_7_:

$$\begin{array}{cc} g=g(y_{2},y_{3},\cdots ,y_{7}) & \varphi =\varphi (y_{2},y_{3},\cdots ,y_{7}) \end{array}$$

The uncertainty matrix of the vector [gφ] is:

$$\left[\begin{array}{cc} u^{2}\;(g) & u\;(g,\varphi )\\ u\;(g,\varphi ) & u^{2}\;(\varphi ) \end{array}\right]=J_{2}\cdot U_{y}\cdot J_{2}^{T}$$

where J_2_ is the Jacobian matrix of the functions *g* = *g*(*y*_2_, *y*_3_, ⋯, *y*_7_) and φ = φ (*y*_2_, *y*_3_, ⋯, *y*_7_), and is given by:

$$J_{2}=\left[\begin{array}{cccc} \partial g/{\partial y}_{2} & \partial g/{\partial y}_{3} & \cdots & \partial g/{\partial y}_{7}\\ \partial \varphi /{\partial y}_{2} & \partial \varphi /{\partial y}_{3} & \cdots & \partial \varphi /{\partial y}_{7} \end{array}\right]$$

and the partial derivatives of matrix J_2_ are evaluated numerically.

For example, at frequency *f* = 47.7 kHz, we obtain the following results:

$$\begin{array}{ccc} g=-12.1\;\text{dB} & \varphi =78.9{}^{\circ} & r(g,\varphi )=\frac{u(g,\varphi )}{u(g,)\cdot u(\varphi )}=+0.54\\ u(g)=0.48\;\text{dB}\; & u(\varphi )=1.3{}^{\circ} & \end{array}$$

**Figure 2.**
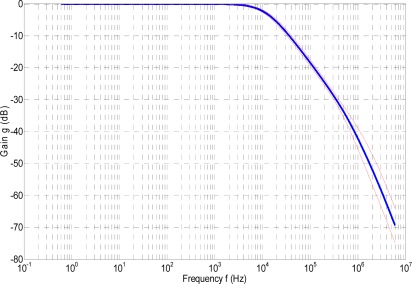
Gain *g* (dB) vs. frequency *f* (Hz).

**Figure 3.**
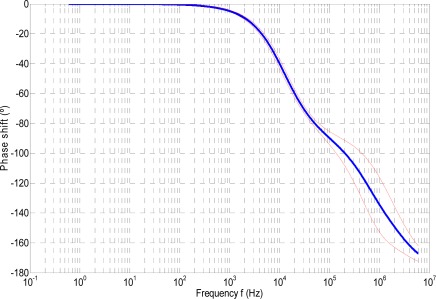
Phase shift φ(°) vs. frequency *f* (Hz).

Figures 2 and 3 show in thick-blue lines the gain g and the phase shift φ of the RPC versus frequency, respectively. The thin-red lines represent the upper and lower boundaries of the expanded uncertainty interval. Expanded uncertainties has been evaluated at a level of confidence of approximately 95%, using a coverage factor of *k* = 2 [25].

#### 4.3.2. Cut-Off Frequency

Another important parameter is the cut-off frequency *f_c_*. For the case under analysis, from the frequency response shown in Figures. 2 and 3, it can be seen that, as the first zero is located between the second and the third pole and the second zero is located right after the forth pole, the cut-off frequency *f_c_* depends mainly on the denominator of *T*_2_(*s*).

In this paper, the cut-off frequency *f_c_* was determined numerically in the frequency range shown in Figures. 2 and 3, and its partial derivatives with respect to y_4_, y_5_, ⋯, y_7_ were determined numerically as well.

In order to be consistent with the above statements, for the analysis, the partial derivatives of *f_c_* with respect to *y*_2_ and *y*_3_ were assumed to be equal to zero. Therefore, the standard uncertainty of the cut-off frequency, *u*(*f_c_*), was calculated as follows:

$$u^{2}(f_{c})=\left[\begin{array}{cccc} \frac{{\partial f}_{c}}{{\partial y}_{4}} & \frac{{\partial f}_{c}}{{\partial y}_{5}} & \frac{{\partial f}_{c}}{{\partial y}_{6}} & \frac{{\partial f}_{c}}{{\partial y}_{7}} \end{array}\right]\cdot \left[\begin{array}{cccc} u^{2}(y_{4}) & r_{45}u(y_{4})u(y_{5}) & r_{46}u(y_{4})u(y_{6}) & r_{47}u(y_{4})u(y_{7})\\ r_{54}u(y_{5})u(y_{4}) & u^{2}(y_{5}) & r_{56}u(y_{5})u(y_{6}) & r_{57}u(y_{5})u(y_{7})\\ r_{64}u(y_{6})u(y_{4}) & r_{65}u(y_{6})u(y_{5}) & u^{2}(y_{6}) & r_{67}u(y_{6})u(y_{7})\\ r_{74}u(y_{7})u(y_{4}) & r_{75}u(y_{7})u(y_{5}) & r_{76}u(y_{7})u(y_{6}) & u^{2}(y_{7}) \end{array}\right]\cdot \left[\begin{array}{c} \frac{{\partial f}_{c}}{{\partial y}_{4}}\\ \frac{{\partial f}_{c}}{{\partial y}_{5}}\\ \frac{{\partial f}_{c}}{{\partial y}_{6}}\\ \frac{{\partial f}_{c}}{{\partial y}_{7}} \end{array}\right]$$

Finally, the results are the following:

$$f_{c}=12,0\;\text{kHz}\;\;\;\;u(f_{c})=0.70\;\text{kHz}\;\;\;\;U_{95\%}(f_{c})=1.4\;\text{kHz}$$

where the expanded uncertainty expanded *U*_95%_(*f_c_*) has been evaluate at a level of confidence of approximately 95%, using a coverage factor of *k* = 2 [25].

## 5. Conclusions

In this paper, the uncertainty of the transfer function of a RPC has been estimated in accordance with the Guide to the Expression of Uncertainty in Measurement of the Organization for Standardization. The RPC transfer function has been described through seven parameters and the uncertainty and correlation coefficients of these parameters have been estimated as well. Also, it has been shown that other parameters such as the gain, phase margin and the cut-off frequency can be estimated along with their respective uncertainties taking into consideration the information given by the RPC transfer function.

## Figures and Tables

**Table 1.** Minimum, typical, maximum value and standard uncertainty of the input quantities.

**Input quantity**	**MIN**	**TYP**	**MAX**	**Standard uncertainty**
C_j_	522 pF	580 pF	638 pF	24 pF
R_j_	374 MΩ	416 MΩ	457 MΩ	17 MΩ
R_s_	5.31 Ω	5.90 Ω	6.49 Ω	0.24 Ω
R_1_	900 Ω	1000 Ω	1100 Ω	41 Ω
R_2_	90.0 Ω	100.0 Ω	110.0 Ω	4.1 Ω
R_3_	90.0 kΩ	100.0 kΩ	110.1 kΩ	4.1 Ω
R_4_	19.87 kΩ	22.08 kΩ	24.29 kΩ	0.90 kΩ
R_i_	15.0 MΩ	50.0 MΩ	55.0 MΩ	2.0 MΩ
*C_i_*	0 pF	0 pF	0 pF	0 pF
A_0_	106 dB	114 dB	125.4 dB	1.46 dB
ω_T_	0.80π Mrad/s	1.20π Mrad/s	1.30π Mrad/s	0.15 Mrad/s
σ	121.1 mA/W	134.5 mA/W	148.0 mA/W	5.5 mA/W

**Table 2.** Parameter estimation, standard uncertainty and relative standard uncertainty.

**Parameter**	**Parameter estimation**	**Standard uncertainty**	**Relative standard uncertainty**
y_2_	0.0053	0.0016	0.30
y_3_	0.42	0.12	0.29
y_4_	0.148	0.029	0.20
y_5_	12.2	2.0	0.17
y_6_	55.9	8.0	0.14
y_7_	50.3	2.9	0.057
